# Beyond traditional proxies: the contribution of leisure to cognitive reserve and dementia

**DOI:** 10.3389/fnagi.2025.1600798

**Published:** 2025-09-18

**Authors:** Silvia Ottaviani, Luca Tagliafico, Stefania Peruzzo, Marta Ponzano, Alessio Signori, Alessio Nencioni, Fiammetta Monacelli

**Affiliations:** ^1^Section of Geriatrics, Department of Internal Medicine and Medical Specialties (DIMI), University of Genoa, Genoa, Italy; ^2^IRCCS Ospedale Policlinico San Martino, Genoa, Italy; ^3^Department of Health Sciences - Section of Biostatistics, University of Genoa, Genoa, Italy

**Keywords:** gender medicine, leisure acitivites, cognitive resilience, CRI, sex difference, Alzheimer’s disease

## Abstract

**Introduction:**

Cognitive reserve (CR) is a multidimensional construct based on lifelong engagement in cognitively stimulating domains, including education, occupation and leisure activities, that plays a crucial role in mitigating the presentation of dementia. To date, the contribution of each CR subdomain in the development of dementia is under-investigated. This study is aimed at assessing the association of CR subdomains with cognitive status, accounting for sex and age in an old-age population.

**Methods:**

317 older adults were recruited with a diagnosis of subjective cognitive impairment, mild cognitive impairment, and dementia due to Alzheimer’s disease or mixed-type dementia. Cognitive Reserve Index questionnaire (CRIq) was used to assess CR. Patients were stratified based on sex and dementia staging (CDR). Significant variables from univariate analysis entered a multivariate ordinal regression model, using CDR as the dependent variable.

**Results:**

The results showed that the leisure activities subdomain was the main determinant of cognitive status (OR 0.90, 95%CI 0.82–1.00, *p* = 0.003); CR and sex did not show any interaction.

**Discussion:**

Unlike education and occupation, leisure activities may be considered a lifelong, dynamic contributor to CR. These findings highlight the importance of refining CR assessment, with particular attention to leisure activities as a potentially modifiable target for dementia prevention.

## Introduction

Ageing involves significant changes in an individual’s physiological reserve that may account for a decline in physical, social and cognitive function. In this context, dementia is a key relevant medical and social challenge, influenced by intrinsic and environmental-based factors and leading to highly individualized variation in disease clinical expression between individuals. So far, the implementation of preventative approaches, based on the systematic prevention of modifiable risk factors, is largely emphasized, accounting for 40% of preventable worldwide cases (Livingston et al., 2024).

Namely, Cognitive Reserve (CR) is a cumulative construct shaped by lifelong engagement in cognitively stimulating activities, including education, work, and leisure activities (Stern, 2009). CR can be defined as “*adaptability that helps explain the differential susceptibility of cognitive abilities or daily functioning to brain ageing”* (Stern et al., 2020). The impact of CR on cognitive aging displays a threshold model (Lövdén et al., 2020), where higher educational and occupational attainment, as well as intellectual engagement, contribute to cognitive skills that persist into later life, delaying the onset of dementia symptoms (Soldan et al., 2017; Kato et al., 2022). This aligns with findings that individuals with higher CR can better tolerate neurodegenerative burden before exhibiting cognitive impairment or dementia (Van Loenhoud et al., 2022; Stern, 2012). Recent advancements in CR analysis, including EEG, eye tracking, and neuroimaging techniques, hold the promise to unveil neural mechanisms of resilience (Menardi et al., 2018), providing a non-invasive real-time assessment of brain activity (Medeiros et al., 2024), enhancing emotional recognition and the timely detection of cognitive impairment (Jiang et al., 2019; Tokushige et al., 2023).

Dementia is a leading cause of mortality in women in high-income countries with a faster and more severe clinical progression compared to men (Stern, 2009; Stern et al., 2020). Emerging evidence underscores that both biological and social determinants may shape sex differences in cognitive resilience and in the development of dementia (Emrani and Sundermann, 2025). Understanding such differences may allow for addressing peculiarities in terms of pathophysiological biomarkers, disease progression, treatment response, and targeted lines of prevention.

Recently, Arenaza-Urquijo et al. suggested that women tend to experience a more rapid cognitive decline as dementia progresses, potentially due to faster rates of amyloid and tau accumulation (Arenaza-Urquijo et al., 2024), as reported in prior studies, addressing the need for further investigation on the role of sex differences in modulating CR.

In the context of the operationalization of CR, there is no established gold standard (Kartschmit et al., 2019; Nogueira et al., 2022). While education is commonly used as a proxy, other indicators such as occupational attainment (Hakiki et al., 2021), reading ability (O'Shea et al., 2015), intelligence quotient (Alty et al., 2023), and composite measures have also been proposed (Nucci et al., 2012) to reflect the individual CR. The multidimensional Cognitive Reserve Index Questionnaire (CRIq) (Nucci et al., 2012) has been developed to integrate three distinct CR subdomains—education, occupational activity, and leisure time—and has been cross-culturally validated in multiple languages and different populations, including older adults (Kartschmit et al., 2019; Nogueira et al., 2022; Ottaviani et al., 2024).

To date, studies investigating sex differences applied to the construct of CR are suboptimal and, similarly, there are no in-depth analyses on which subdomain of the multidimensional construct of CR might play a key relevant role in predicting the risk of dementia (Rouillard et al., 2017).

Drawing upon available scientific background, the present study is aimed at assessing the association of CR subdomains with cognitive status, while accounting for sex and age, in an old-age outpatient population.

## Materials and methods

This cross-sectional study included 317 consecutive patients attending the outpatient geriatric memory clinic of the IRCCS San Martino Polyclinic Hospital (Genoa, Italy), from September 2022 to March 2023. The study was conducted in accordance with the Declaration of Helsinki, and approved by the Institutional Review Board of the University of Genoa (protocol code 54, 2024-06-12).

Inclusion criteria were: age 65 years and older, diagnosis of Subjective Cognitive Impairment [SCI (Jessen et al., 2014)], Mild Cognitive Impairment [MCI (American Psychiatric Association, 2013)] or dementia of Alzheimer’s type, and mixed dementia, according to DSM-V diagnostic criteria (American Psychiatric Association, 2013).

Exclusion criteria were: diagnosis of major psychiatric disorders or psychosis; diagnosis of dementia of other neurodegenerative types (e.g., dementia with Lewy bodies, vascular dementia); diagnosis of major depression (American Psychiatric Association, 2013); diagnosis of incident delirium (American Psychiatric Association, 2013).

Cognitive status was evaluated at baseline using the Mini-Mental State Examination [MMSE (Frisoni et al., 1993)], and the Clinical Dementia Rating Scale [CDR (Morris, 1993)] was used to stage dementia severity. CR was measured using CRIq (Nucci et al., 2012). Supplementary material S1 contains the full details of the CRIq.

Throughout this study, we will use the term “*sex*,” while acknowledging that many disease-related differences arise from a complex interplay of biological and sociocultural factors that are difficult to disentangle; it is crucial to recognize that this binary framework does not fully capture the complexity of sex and gender (Emrani and Sundermann, 2025).

### Statistical analysis

A descriptive analysis of patients’ clinical phenotype was conducted and the stratification based on sex and cognitive status was performed. Namely, based on CDR staging, patients who scored zero were categorized as cognitively intact; patients who scored 0.5, 1, 2, and 3, were staged as having MCI, mild stage dementia, moderate stage dementia and advanced stage dementia, respectively. The appropriate Mann–Whitney test was applied.

All variables with *p*-value <0.10 at the univariate analysis were selected to enter a multivariate ordinal regression model (Ranganathan et al., 2017) with CDR score (categorized as follows: 0 vs. 0.5 vs. 1–4) as the dependent variable. The independent variables included age, CRIq subitems, and disease duration, with sex added as an adjustment variable. To explore the relationship between sex and CR, all interaction terms between CRIq subitems and sex were also tested.

An additional sex-adjusted univariate and multivariate linear regression model using the MMSE score as the dependent variable, and a logistic regression model with binary CDR (0 vs. 0.5–4) as the dependent variable were performed as part of the sensitivity analyses, as well as a non-sex-adjusted ordinal regression model.

All reported analyses were run by RStudio (Version 2022.07) and a two-sided *α* less than 0.05 was considered statistically significant.

## Results

Patients had a mean age of 84 years (IQR 7, range 67–95), with a higher prevalence of females (67%)(Table 1). The median MMSE score was 23.3, with no sex differences. Namely, 80 patients had a diagnosis of SCD (25.2%), 77 patients had a diagnosis of MCI (24.3%), and 160 patients received a diagnosis of dementia (50.5%). The median disease duration was 1.18 years.

**Table 1:** Patients’ clinical phenotype.

Total sample *N* = 317	*N* (%), median (IQR)
Age	84 (7)
Females	213 (67%)
CDR	
0	80 (25%)
0.5	77 (24%)
1–4	160 (51%)
MMSE	23.3 (8.3)
Disease duration (years)	1.18 (2.69)
CRIq	
Total	97 (27)
Education	100 (19)
Occupation	92 (29)
Leisure	103 (35)

CDR, Clinical Dementia Rating scale; MMSE, Mini Mental State Examination; CRIq, Cognitive Reserve Index questionnaire.

Women showed lower scores across all CRIq subdomains (education, work, leisure) (Table 2), indicating a sex difference.

**Table 2:** Patients’ clinical phenotype as for cognitive status (CDR 0 vs. 0.5–4) and as for sex.

Variables	CDR = 0 (median)	CDR = 0.5–4 (median)	*p* value	Female (median)	Male (median)	*p* value
	*N* = 80	*N* = 237		*N* = 213	*N* = 104	
Age	83	83.4	0.471	83.2	83.5	0.51
MMSE	28	21.4	<0.001	23.2	23.3	0.747
CRIq						
Total	99	96	0.084	94	106	<0.001
Education	102	99	0.125	98	103	0.036
Occupation	93.5	92	0.711	84	97	<0.001
Leisure	107	101	0.023	98	107	0.019

CDR: Clinical Dementia Rating scale; MMSE: Mini Mental State Examination; CRIq: Cognitive Reserve Index questionnaire.

Based on CDR staging, patients with MCI or dementia (CDR score of 0.5 or higher) were more likely to exhibit lower CRIq leisure subdomain scores compared to cognitively intact individuals (CDR score of 0), suggesting a lower lifetime engagement in leisure activities.

After adjusting for age, disease duration, and sex, the ordinal regression model showed an association between the CRIq leisure score and cognitive status (OR = 0.90, 95%CI: 0.82–1.00, *p* = 0.003). No statistically significant interactions were found between sex and any of the CRIq domains (Table 3). Results were consistent in a sensitivity analysis performed without adjusting for sex (Supplementary material S2).

**Table 3:** Multivariate ordinal regression model with CDR score (0 vs. 0.5 vs. 1–4) as the dependent variable, including interaction terms between sex and CR subdomains.

Variables	Univariate model		Multivariate model	
	OR (95% CI)	*p* value	OR (95% CI)	*p* value
Age	1.07 (1.03–1.11)	0.011	1.05 (1.01–1.10)	0.018
Sex (ref: female)	0.82 (0.52–1.27)	0.371	0.64 (0.38–1.07)	0.089
Disease duration (+ 1 year)	1.21 (1.09–1.36)	<0.001	1.19 (1.06–1.34)	0.003
CRIq education (+10 pt)	0.90 (0.74–1.00)	0.054	1.00 (0.98–1.10)	0.997
CRIq occupation (+10 pt)	0.90 (0.82–1.00)	0.020	1.00 (0.90–1.10)	0.371
CRIq leisure (+10 pt)	0.82 (0.74–0.91)	<0.001	0.90 (0.82–1.00)	0.003
Sex*CRIq education	1.00 (0.97–1.04)	0.810		
Sex*CRIq occupation	1.01 (0.99–1.04)	0.360		
Sex*CRIq leisure	0.99 (0.97–1.01)	0.438		

CRIq, Cognitive Reserve Index questionnaire.

The linear regression model (MMSE score as the dependent variable) supported the association of CRIq leisure and cognitive status (*β* = 0.08, 95%CI: 0.02–0.14, *p* = 0.006), as did the multivariate logistic regression model with the binarized CDR score as outcome (OR = 0.91, 95%CI: 0.82–1.00, *p* = 0.046) (see Supplementary materials S3 and S4, respectively).

## Discussion

In the last decades, growing evidence has investigated the role of CR in older adults with different types of cognitive decline, mainly pertaining to education and occupation. However, to the best of our knowledge, this is among the few studies to investigate the role of CR subdomains in mediating the association with cognitive status in older adults. We sought to analyze differences in CR subdomains and their influence on cognitive status in a real-world cohort of older adults.

Based on our results, women exhibited lower overall CRIq scores, indicating a sex-related disadvantage in the accumulation of CR, supporting existing literature (Nucci et al., 2012; Maiovis et al., 2016; Slavić et al., 2022). Such findings might be attributed to the fact that women have historically had fewer opportunities to build CR due to limited access to education and fewer opportunities for employment complexity (Subramaniapillai et al., 2021). Previous research has shown that older women show relatively better cognitive performance in countries with more egalitarian gender-role attitudes, an effect that is partially mediated by differences in education and labor force participation (Bonsang et al., 2017).

Our results also showed that leisure time is a crucial component of CR and it is associated with dementia staging, even after adjusting for age, disease duration and sex. In line with that, Del Ser et al. showed that leisure activities were significantly associated with cognitive function, accounting for more than 20% of the variance (Del Ser et al., 2023). Similarly, Leung et al. found that higher leisure activity levels were associated with a lower risk of MCI (Leung et al., 2024), and Verghese et al. underscored that leisure activities showed the main protective role on the risk of developing dementia compared to physical activity (Verghese et al., 2003), although reverse causality was not excluded.

Notably, Pa et al. (2022) demonstrated that leisure activities contributed to the maintenance of cognitive processing speed in both men and women, while the contribution of CR to sustaining memory may be subject to sex-specific differences.

It could be hypothesized that, unlike education and occupation, which may be considered static constructs, as they are typically established earlier in life and are no longer modifiable in old age, leisure activities could be promoted throughout life, offering a dynamic enhancement of CR and cumulating until late life. Moreover, leisure time includes several activities and also takes into account years of practice. Social engagement and social participation—such as maintaining interpersonal relationships, participating in community activities, and engaging in group-based learning or recreational programs—may offer a dual benefit in shaping CR. On one hand, it promotes cognitive functioning by stimulating communication, problem-solving, and cognitive flexibility (Evans et al., 2019; Krueger et al., 2009). On the other hand, it mitigates loneliness and social vulnerability, ascertained risk factors for dementia.

No significant interaction was found between CR and sex, suggesting that sex does not intervene to mediate the relationship between CR and cognitive status. In other words, once CR is accumulated, its protective effects on cognitive status appear to be similar for both sexes. This could imply that, despite differences in CRIq scores, men and women benefit from CR in comparable ways; what makes a difference is the accumulation of such baggage, which appears strongly genderized. Alternatively, the lack of interaction may be the result of limited statistical power or unmeasured factors (e.g., social support, genetic predisposition) that warrant further investigation.

Limitations include the monocentric setting and the cross-sectional nature of the study design, which may reduce the generalizability of the findings.

Strengths include the analysis of the CR construct in a real-world old-age population using a validated CR multidimensional instrument such as the CRIq.

Longitudinal studies are warranted to investigate interaction or causation terms and to integrate biomarkers from neuroimaging, plasma/cerebrospinal fluid, and functional imaging, as well as APOE4 status, to refine our understanding of the biological basis of CR. For instance, recent advances in neuroimaging have underscored the value of multimodal approaches in exploring the neural basis of CR. Martínez et al. demonstrated through magnetoencephalography that individuals with higher CR exhibited more efficient functional network configurations during memory tasks, requiring fewer and more optimally distributed connections (Martínez et al., 2018). Other studies have identified specific brain network features related to CR using PET and fMRI (Lee et al., 2019), while dynamic network models based on spatio-temporal graph theory offer promising tools for detecting topological biomarkers of CR (Zhu et al., 2024).

Moreover, since CRIq categorizes activities based on their frequency (daily, monthly, yearly) rather than their nature (e.g., intellectual, social, or physical), more structured analysis may help establish whether particular types of activity, such as social leisure, contribute in a different manner to CR.

In conclusion, this study may be a platform for developing future research aimed at integrating biological, clinical, and socio-cultural factors to provide a more comprehensive understanding of CR and its role in shaping cognitive status in older age. Our data may support adopting a dementia care approach that emphasizes the role of CR—particularly through leisure activities—which should be considered a lifelong modifiable risk factor.

## Data Availability

The raw data supporting the conclusions of this article will be made available by the authors, without undue reservation.
